# Influence of structural reinforcements on the twist-to-bend ratio of plant axes: a case study on *Carex pendula*

**DOI:** 10.1038/s41598-021-00569-z

**Published:** 2021-10-27

**Authors:** Steve Wolff-Vorbeck, Olga Speck, Thomas Speck, Patrick W. Dondl

**Affiliations:** 1 Department of Applied Mathematics, University of Freiburg, Hermann-Herder-Str. 10, 79104 Freiburg, Germany; 2 Plant Biomechanics Group @ Botanic Garden, Faculty of Biology, University of Freiburg, Schänzlestraße 1, 79104 Freiburg, Germany; 3 Cluster of Excellence livMatS @ FIT-Freiburg Center for Interactive Materials and Bioinspired Technologies, University of Freiburg, Georges-Köhler-Allee 105, 79110 Freiburg, Germany

**Keywords:** Plant sciences, Biomaterials, Applied mathematics

## Abstract

During biological evolution, plants have developed a wide variety of body plans and concepts that enable them to adapt to changing environmental conditions. The trade-off between flexural and torsional rigidity is an important example of sometimes conflicting mechanical requirements, the adaptation to which can be quantified by the dimensionless twist-to-bend ratio. Our study considers the triangular flower stalk of *Carex pendula*, which shows the highest twist-to-bend ratios ever measured for herbaceous plant axes. For an in-depth understanding of this peak value, we have developed geometric models reflecting the 2D setting of triangular cross-sections comprised of a parenchymatous matrix with vascular bundles surrounded by an epidermis. We analysed the mathematical models (using finite elements) to measure the effect of either reinforcements of the epidermal tissue or fibre reinforcements such as collenchyma and sclerenchyma on the twist-to-bend ratio. The change from an epidermis to a covering tissue of corky periderm increases both the flexural and the torsional rigidity and decreases the twist-to-bend ratio. Furthermore, additional individual fibre reinforcement strands located in the periphery of the cross-section and embedded in a parenchymatous ground tissue lead to a strong increase of the flexural and a weaker increase of the torsional rigidity and thus resulted in a marked increase of the twist-to-bend ratio. Within the developed model, a reinforcement by 49 sclerenchyma fibre strands or 24 collenchyma fibre strands is optimal in order to achieve high twist-to-bend ratios. Dependent on the mechanical quality of the fibres, the twist-to-bend ratio of collenchyma-reinforced axes is noticeably smaller, with collenchyma having an elastic modulus that is approximately 20 times smaller than that of sclerenchyma. Based on our mathematical models, we can thus draw conclusions regarding the influence of mechanical requirements on the development of plant axis geometry, in particular the placement of reinforcements.

## Introduction

### Morphology, anatomy and biomechanics of plants

Functional morphology of plants includes morphology, anatomy and biomechanics of entire plant organs and single plant tissues^1^. In the following we consider the biomechanics of plant axes, in particular with respect to their reaction to bending and torsional loads. In general, flexural rigidity and torsional rigidity are composed variables that combine material and geometrical properties. Only in the simple setting of homogeneous materials with nearly circular geometry, flexural rigidity (*EI*) is determined by the bending elastic modulus (*E*) and the axial second moment of area (*I*), whereas torsional rigidity (*GJ*) is approximately given by the torsional modulus (*G*) and the polar second moment of area (*J*). The bending and torsional moduli (SI unit: N m$^{-2}$ or Pascal) specify the stress–strain relationship as a measure for rigidity in the linear-elastic range, whereby mechanical stress is defined as the applied force per unit area and strain is understood as the displacement relating to a reference condition. The geometrical properties, namely the axial and polar second moments of area (SI unit: m$^{4}$), reflect the way in which the points of an area are distributed in relation to a bending or torsional neutral plane or torsional axis, respectively^2^. In this context, the dimensionless twist-to-bend ratio (*EI*/*GJ*) is particularly useful, as it provides information about the trade-off between rigidity in bending and in torsion. Bending and torsion are two different types of loading to which plants are exposed by their own weight and additional loads such as wind, flowers, fruits, snow, and animals sitting or climbing on the plants. A high twist-to-bend ratio reflects high bending rigidity combined with low torsional stiffness. High bending rigidity guarantees that leaves or flower stalks stand mostly upright, even when carrying large top loads (e.g., leaf blades, flowers and fruits). A high torsional flexibility, in the sense of the inverse of rigidity, allows streamlining in the wind, so that even plant parts with a large surface area (e.g. leaf blades) turn out of the wind, thereby reducing drag and enabling the plant to withstand higher wind loads without damage. During biological evolution, plants have developed morphological, anatomical and biomechanical properties that are optimised to sometimes conflicting demands of bending and torsion.

Herbaceous plants have a variety of tissues such as epidermis, parenchyma, vascular bundles, collenchyma and sclerenchyma that require different amounts of metabolic energy to be built. The formation of fibers as a reinforcement of plant axes is particularly energy-intensive because, in contrast to parenchyma and epidermis, thick cell walls must be formed and, in the case of sclerenchyma, the macromolecule lignin must be synthesised, which serves as an impregnation of their cell walls. Mechanical tests on whole biological plant axes can determine their overall mechanical properties, but not the individual in situ contribution of particular tissues. Within the framework of this interdisciplinary collaboration between scientists from the fields of plant biomechanics and applied mathematics, we have developed models and simulations that enable experiments in which the influence of in situ (fibre) reinforcements can be analysed. From the results of our experiments, in which either the two-dimensional arrangement or the mechanical properties of the individual tissues have been changed, we can draw conclusions about the in situ influence of individual tissues on the overall mechanical performance of the respective plant axis.

Plants differ in their so-called general body plan, which is a set of morphological features common to many members of a phyllum^3^. This includes, for example, geometry, shape and size of their plant axes^4^. Thus, the cross-sections of the petioles of the elephant ear (*Caladium bicolor* (Aiton.) Vent.; hereafter *C. bicolor*) are massive and circular^5^, the stems of the giant reed (*Arundo donax* L.) are hollow and circular^6^, the branches of the prickly pear (*Opuntia ficus-indica* (L.) Mill.; hereafter *O. ficus-indica*) are elliptical^7^, the flower stalks of the motherwort (*Leonurus cardiaca* L.; hereafter *L. cardiaca*) are square^8^ and the flower stalks of the drooping sedge (*Carex pendula* Huds.; hereafter *C. pendula*) are triangular^2^. There are a number of ways in which plants can increase their twist-to-bend ratio. Independent of the geometry of the cross-section, the polar second moment of area *J* is the sum of the axial second moments of area in x-direction $I_{x}$ and in y-direction $I_{y}$. Consequently, the ratio of *I*/*J* cannot exceed 1.0. This is different if the torsional constant *K* is calculated instead of the polar second moment of area^9^. The torsional constant *K* can be considerably smaller than *J* and thus leads to ratios of $I/K > 1$. However, if the *I*/*J* or *I*/*K* ratios are below 1, high twist-to-bend ratios must be attributed to high ratios of elastic and torsional modulus (*E*/*G*)^10^. The above considerations are only valid if one considers homogeneous elastic materials, where the torsional rigidity is given by the product *GK* (or its approximation *GJ*). Many plants, however, should be regarded as fibre-reinforced materials systems defined by the three-dimensional arrangement of their tissues, each of them with characteristic material properties. This makes a more detailed modeling necessary, where the torsional rigidity is computed by solving an appropriate partial differential equation. For more information on the approach used here, see section “Mathematical models”. Additionally, biological structures are not only anatomically inhomogeneous and mechanically anisotropic, but also possess a spatial and temporal heterogeneity because of their growth and reaction capacity^4^.

Plant tissues differ in their anatomy, biomechanics and functions. Many of their properties are related to the presence of a cell wall and the large vacuole within the cell. The epidermis is the outermost tissue of the plant and consists of a single layer of cells covered by a cuticle. The epidermal tissue (including the cuticle) protects against water loss and regulates gas change. The periderm is a secondary covering composed of multiple layers containing cork cells. It sometimes covers the epidermis and protects the stem from desiccation and pathogen attack. The parenchyma consists of living thin-walled cells with multiple functions. As a result of a high turgor pressure inside the vacuoles of these cells, the parenchyma can contribute to flexural and torsional rigidity and holds under mechanical loading the specialised strengthening tissues in place. Chlorenchyma cells contain many chloroplasts for photosynthesis. The aerenchyma is a spongy tissue that allows gas exchange. Sclerenchyma fibres are dead cells characterised by thick and lignified cell walls. Collenchyma fibres are living cells with thick and non-lignified cell walls. The sclerenchyma and collenchyma belong to the strengthening tissues that provide load-bearing support for the plant and its organs. The vascular bundles are part of the transport system for water (xylem) and sugars (phloem). Tracheids and vessel elements of the xylem are dead and possess thick-walled and lignified cell walls, which also provide mechanical support.

### Modelling of biological materials systems

Plants are multifunctional structures whose diverse functions are anchored to six hierarchical levels (molecule, organelle, cell, tissue, organ, plant). In this study, we focus on the tissue level and the modelling of the influence of the two-dimensional tissue distribution on the mechanical performance of the entire plant axis. Experiments and findings within the models go far beyond those on plants. For example, the number of fibre bundles can be increased or reduced, even though the amount of material remains the same if the dimensions (diameter) of the bundles change accordingly. Furthermore, in the simulation, lignified sclerenchyma fibres can be replaced by non-lignified collenchyma fibres, whereby the elastic modulus of the sclerenchyma fibres is one order of magnitude higher than that of the collenchyma fibres. In contrast, the non-lignified parenchyma, which often takes up the largest part of the plant cross-section, and the epidermis, a dermal tissue comprising a single layer of cells, have very low elastic moduli, which are on average one to three orders of magnitude smaller than those of the collenchyma fibres^4,8,11–13^. The simulation also allows us to change a non-lignified single-layered epidermis into a corky multi-layered peridermal tissue, which is at least 10-times as stiff as the epidermis (unpublished data).

A cornerstone of plant biomechanics is the performance of mechanical tests under a variety of applied loads such as tension, bending, compression and torsion, both in the linear-elastic range and up to ultimate strength, i.e. at failure. Data of the mechanical tests and the corresponding geometrical properties in terms of size, shape and tissue arrangement are necessary to develop analytical and numerical models. The models not only enable a deeper understanding of the functional principles of the plant model, but are also an indispensable precondition for the transfer of knowledge to technical developments, because models represent a common language for natural scientists, mathematicians, materials scientists and engineers^14^. According to the biomimetic approaches of the “biology push process” or “bottom-up approach” and the “technology pull process” or “top-down approach”, every successful biomimetic product has to go through a step of abstraction^15^.

Both analytical and numerical modelling have their intrinsic advantages and short comings when they are used to improve our understanding of the form-structure-function relationship of the biological models or to facilitate the transfer into biomimetic applications. Analytical models, for example, allow fast predictions about the influence of a variety of structural, morphological and/or anatomical changes in a plant organ on its mechanical properties and also enable the inclusion of predictions about non-existing intermediate or extreme forms in the analysis. Similar to the requirements of a “closed” mathematical description, as desired in analytical models, the number of variables and the boundary conditions have to be limited, and a careful simplification in the description of the biological model is typically necessary. The reduction to a few characterising variables allows, on the one hand, a (much) faster analysis of the biological models. On the other hand, this procedure unfortunately includes dangers of oversimplification and of decisive variables being overlooked. The potential of analytical models for an in-depth understanding of the biological model and the transfer to a technical application was demonstrated in the following studies: mechanically driven self-sealing function of the leaves of *Delosperma cooperi*^16^, tuyere surfaces of metals inspired by lotus leaves^17^, and modelling of stomatal density response to atmospheric CO$_{2}$^18^.

Numerical models (often) permit the precise description of form and structure of a biological model, but frequently need a plethora of variables. The measurement of all of these variables to the accuracy needed for numerical models is mostly extremely time-consuming (and sometimes even impossible). Moreover, numerical models are only of limited value for the inclusion and prediction of non-existing intermediate or extreme forms in the analysis. The latter is especially true as far as changes in form and structure are involved, whereas variations in mechanical variables are typically easy to include in numerical models. The potential of numerical models for a deeper understanding of the biological model and the transfer to a technical application was demonstrated in the following studies: hydraulically driven self-sealing function of the leaves of *Delosperma cooperi*^19^, the biomimetic cellular actuator inspired by turgor driven plant movement^20^, and elastic systems in architecture transferred from plant movements^21^.

Now that the advantages and shortcomings of analytical and numerical modelling have been pointed out, it becomes obvious that a combination of both approaches can be considered as the “royal road” of modelling, combining the advantages and avoiding the disadvantages of both approaches. In this work, we have therefore used simplified materials models and have made some geometrical assumptions to be able to focus on the essential variables of the model. The resulting description is too complex to provide a closed form solution but, is simple enough that, by using a finite element approach, we can efficiently analyse a whole range of variable values. Detailed information on the chosen approach is given in section “Mathematical models”.

### Aim of the study

In the study presented, we selected the triangular flower stalk of *Carex pendula* as model system, because to our knowledge, it posses the highest twist-to-bend ratios ever measured for herbaceous plant axes. Therefore, it represents a prime example for testing our models as to their predictive strength. We aim to find answers to the following scientific question: “To what extent do individual tissues such as fibres, vascular bundles, epidermis and parenchyma contribute to the flexural rigidity, the torsional rigidity and thus to the twist-to-bend ratio of a triangular plant axis?” Mathematical calculations have therefore been carried out, based on mechanical and geometrical properties from the literature. In this context, the effect of additional reinforcements on the flexural rigidity, the torsional rigidity and the twist-to-bend ratio have been examined with regard to: (1) the formation of a periderm instead of an epidermis, (2) an increasing number of fibre strands up to an optimum, while keeping their total area in a cross-section constant and (3) the replacement of sclerenchyma fibres by collenchyma fibres, two fibre types that differ notably in their elastic moduli.

## Plant data for modelling

**Figure 1:**
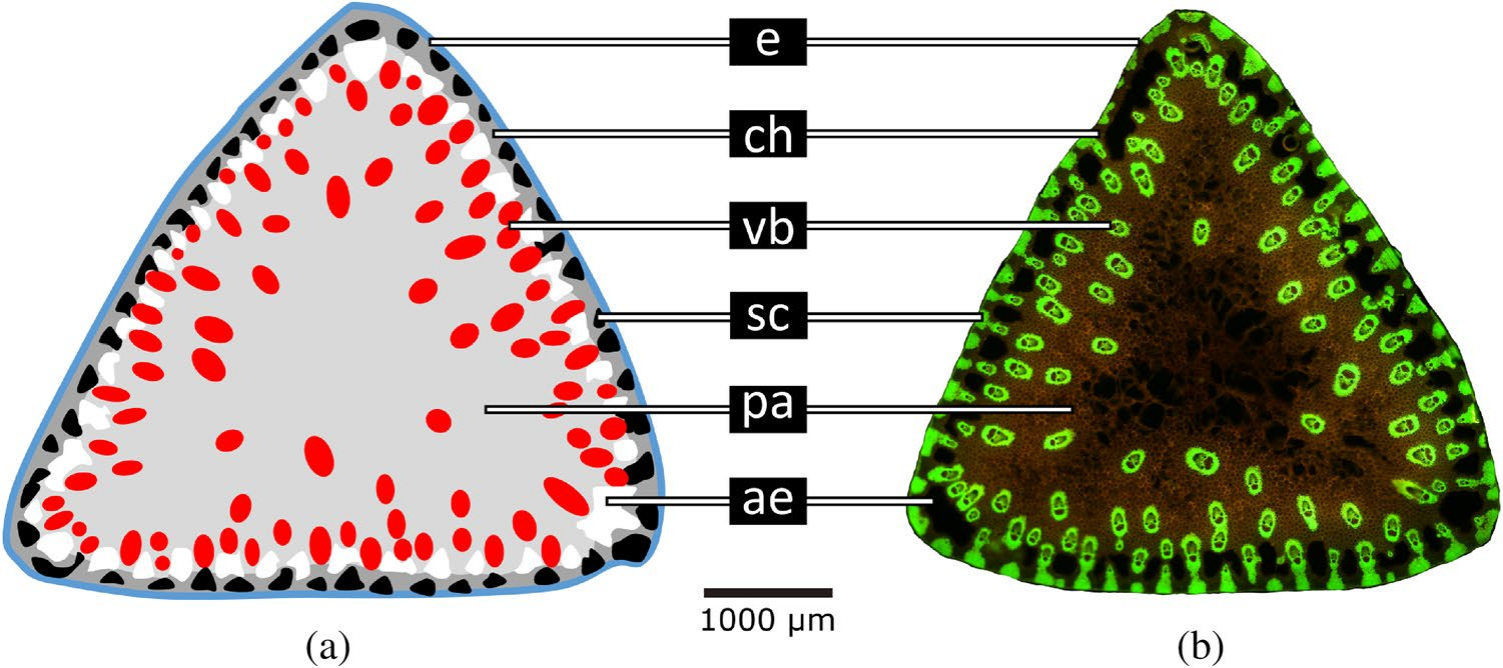
Internodal cross-section of the biological model *Carex pendula*. (**a**) Schematic drawing highlighting the tissues. (**b**) Thin-section stained with acridine orange revealing lignified tissues in bright yellow-green. Abbreviations and colour code: *ae* aerenchyma (white), *ch* chlorenchyma (dark grey, only in the periphery), *e* epidermis (blue), *pa* parenchyma (light grey), *sc* sclerenchyma fibre strands (black), *vb* vascular bundles (red).

*Carex pendula* with its peak values of twist-to-bend ratio is a very suitable model plant for studying the triangular geometry. The necessary data of geometrical and mechanical properties for the mathematical calculations presented below have been collected in previous studies of the Plant Biomechanics Group Freiburg^2,6,8^. Figure 1 presents a stained section and schematic drawing of its flower stalks showing the cross-sectional distribution of tissues. The triangular cross-section shows an outer epidermis (e). Individual lignified sclerenchyma fibre strands (sc) in the periphery and vascular bundles (vb) scattered within the outer half of the cross-section are embedded in a non-lignified ground tissue consisting largely of parenchyma (pa) with interspaced smaller regions of aerenchyma (ae) close to the periphery and a layer of chlorenchyma (ch) directly underneath the epidermis.

Table 1 presents experimental data of the flower stalk of *C. pendula*. Mechanical properties from bending and torsional tests are provided, as are geometrical properties of the entire internode and of individual tissues.

**Table 1:** Geometrical and mechanical properties of the flower stalk of *Carex pendula*.

data	*Carex pendula*
Plant organ	Internode of flower stalk
Cross-sectional geometry	Triangular
Flexural rigidity *EI* (N$\cdot$mm$^{2}$)	$141 \ 873 \pm 61 \ 896$
Bending elastic modulus *E* (N/mm$^{2}$)	$16 \ 132 \pm 3305$
Axial second moment of area *I* (mm$^{4}$)	$9.03 \pm 3.74$
Torsional rigidity *GJ* (N$\cdot$mm$^{2}$)	$825.60 \pm 412.98$
Torsional modulus *G* (N/mm$^{2}$)	$37.90 \pm 11.87$
Polar second moment of area *J* (mm$^{4}$)	$22.09 \pm 9.43$)
Twist-to-bend ratio *EI*/*GJ* (–)	$192.42 \pm 82.53$
4-point bending test/number *n* (/)	15
Torsional test/number *n* (–)	15
Reinforcement fibres	Sclerenchyma
Total area of cross-section (mm$^{2}$)	$7.07 \pm 1.34$
Total area of sclerenchyma (mm$^{2}$)	$1.19 \pm 0.21$
Total area of collenchyma (mm$^{2}$)	–
Total area of vascular tissues (mm$^{2}$)	$2.09 \pm 0.41$
Thickness of epidermis ($\upmu$m)	$14.11 \pm 4.04$
Number of sclerenchyma strands (–)	$56.00 \pm 3.87$
Number of vascular bundles (–)	$77.67 \pm 4.78$
References	Basal internode no. 2 from May^2^

Mean values ± one standard deviation of the mechanical and geometrical properties of the entire internodes and the geometrical properties of individual tissues with reference to the cross-section are presented.

Table 2 shows the elastic modulus of individual tissues such as the epidermis, parenchyma, sclerenchyma fibres, collenchyma fibres and vascular bundles. Elastic moduli were estimated for the respective tissues based on values from the literature and were additionally normalised in relation to the sclerenchyma having the highest value of these elastic moduli. The normalised values given in Table 2 are also included in the mathematical analyses of the influence of fibre reinforcement of plant axes on the twist-to-bend ratio presented below.

**Table 2:** Elastic moduli of individual plant tissues. Literature values ($E_{lit}$) and estimated values ($E_{est}$) for the species *Carex pendula*, *Leonurus cardiaca* and *Opuntia ficus-indica* are provided.

tissue	$E_{lit}$ (MPa)	species	$E_{est}$ (MPa)	$E_{norm}$ (/)
Sclerenchyma*	24,500–45,000^4,11,22^	*C. pendula*	45,000	1.00
Collenchyma	1000–2600^4,11,12^	*L. cardiaca*	2500	0.05555
Vascular bundles	30–840^4,11,13^	*C. pendula*	1000	0.02222
Epidermis + periderm	350–500 (unpubl. data)	*O. ficus-indica*	500	0.01111
Epidermis (non-lignified)	3–250^11,13,23^	*C. pendula*	50	0.00111
Parenchyma and chlorenchyma (non-lignified)	5–100^11^	*C. pendula*	20	0.00044

*Since the elastic modulus of the sclerenchyma had the highest value, it was used as the reference tissue for the calculation of the normalised elastic moduli ($E_{norm}$) used in the mathematical models.

## Mathematical models

In order to describe the influence of fibre reinforcement to the twist-to-bend ratio of a plant, we can consider a 2D-model determining the flexural and torsional rigidity of a beam its cross-section. In our setting, it does not suffice to simply consider the products of Young’s and shear modulus with second and polar moment of area, respectively given by *EI* and *GJ*, as these approximations are only valid for nearly circular cross-sections of homogeneous tissue.

As in our previous work^24^ we use methods from linearized elasticity, which we repeat here for the readers convenience. As we are interested in investigating mechanical properties of the flower stalk of *C. pendula* we describe a plant stem as a long thin elastic rod with domain $B= \Omega \times (0,L)$ of length *L* and simply connected cross-section $\Omega$ remaining constant along the longitudinal axis. We can now consider the domain $\Omega$ as a composite of different materials bounded by a sufficiently regular boundary curve $\partial \Omega$. Further, we assume $L\gg {\text {diam}} \Omega$ and isotropy for the materials involved. Anisotropic effects, viscosity and other time-dependent processes are neglected here since we are only interested in the influence of the cross-sectional geometry and the contained distribution of various materials on the mechanical properties of the stem. For our modelling, we consider *B* fixed at $z=0$ and bending of *B* to be due to an outer normal force on $\Omega$ at $z=L$.

Mora and Müller^25^ have mathematically rigorously derived the flexural (or bending) rigidity by considering the limit of a very slender and long rod, confirming the classical approaches used here. Following this classical theory, see Crandall et al.^26^, flexural rigidity can be deduced from the moment curvature relation

1 $$\begin{aligned} \begin{pmatrix} M_{ x } \\ M_{ y } \end{pmatrix} = \begin{pmatrix} D_{x}&{} D_{xy}\\ D_{xy}&{}D_{y} \end{pmatrix} \cdot \begin{pmatrix} \kappa _{x}\\ \kappa _{y} \end{pmatrix} \end{aligned}$$

where $M_{y},M_{x}$ denote the bending moments applied at the end of the beam ($z=L$), $\kappa _{x}, \kappa _{y}$ denote the curvature in the direction of *x* and *y* respectively and the moments of inertia $D_{x},D_{y}$ and the product of inertia $D_{xy}$ are given by

$$D_{x} = \int\limits_{\Omega } {E(x,y)\hat{x}^{2} } \;{\text{d}}x{\text{d}}y,\;\;D_{y} = \int\limits_{\Omega } {E(x,y)\hat{y}^{2} } \;{\text{d}}x{\text{d}}y,\;\;D_{{xy}} = \int\limits_{\Omega } {E(x,y)\hat{x}\hat{y}} \;{\text{d}}x{\text{d}}y,$$

where we have considered the coordinate system $({\hat{x}},{\hat{y}})$ which has its origin at the centroid of the cross-section $\Omega$, i.e,

$$\begin{aligned} {\hat{y}}= y- \frac{\int \limits _{\Omega } E(x,y) y ~ \mathrm {d}x\mathrm {d}y}{\int \limits _{\Omega } ~ E(x,y)\mathrm {d}x\mathrm {d}y}, \ \ {\hat{x}}= x- \frac{\int \limits _{\Omega } E(x,y) x ~ \mathrm {d}x\mathrm {d}y}{\int \limits _{\Omega } E(x,y) ~ \mathrm {d}x\mathrm {d}y}. \end{aligned}$$

Because of the heterogeneity of $\Omega$ the elastic modulus *E*(*x*, *y*) depends on the cross-sectional coordinates, being piece-wise constant. The maximal and minimal flexural rigidities $D_{\mathrm {max}}$ and $D_{\mathrm {min}}$ along the principal axes are then given by the maximal and minimal eigenvalue of the matrix in (1) leading to

2 $$\begin{aligned} D_{\text {max/min}}= \left( D_{\text {mean}}\pm \sqrt{\frac{(D_{x}-D_{y})^{2}}{4}+D_{xy}^{2}}\right) \end{aligned}$$

with $D_{\text {mean}} = \frac{D_{x}+D_{y}}{2}$. In the following we are concerned with the problem of generating cross-sections with high flexural rigidity. Therefore, we will confine ourselves to the computation of the minimal flexural rigidity $D_{\text {min}}$. High values of $D_{\text {min}}$ then lead to high resistance against bending forces in any direction orthogonal to the flower stalk.

As for the flexural rigidity, the torsional rigidity for an elastic slender rod with domain *B* has also been mathematically rigorously derived by Mora and Müller^25^. In order to describe the torsional rigidity, we thus use St.Venant’s theory of pure torsion of nonhomogeneous elastic beams, which has been employed, among others, by Ecsedi^27^. Torsion is assumed to be due to a moment *T* at the top of *B* and thus the torsional rigidity can be expressed by Prandtl’s stress function $\phi (x,y)$ satisfying

3 $$\begin{aligned} \nabla \cdot \left( \frac{1}{G(x,y)}\nabla \phi \right)&=-2, \ \text {in} \ \Omega , \nonumber \\ \phi&=0 \ \ \text {on} \ \partial \Omega \end{aligned}$$

with the shear modulus *G*(*x*, *y*) depending on the cross-sectional coordinates, being piece-wise constant. By using the stress function $\phi$, the torsional rigidity is then given by

4 $$\begin{aligned} D_{z}= 2 \int \limits _{\Omega } \phi ~ \mathrm {d}x\mathrm {d}y. \end{aligned}$$

In order both to solve Eq. (3) numerically and thus to compute the rigidities in Eqs. (2, 4) we have employed a P1 triangular finite element discretisation of the cross-section $\Omega$. To be precise, $\Omega$ was chosen as an equilateral triangle and discretized using approximately $5\cdot 10^{6}$ triangular elements, thus finely resolving the material heterogeneities due to fibre reinforcement (see, e.g., Fig. 6a). The implementation of this standard finite element method (C++-code) is available in the supplementary material.

## Effect of reinforcements on the twist-to-bend ratio

**Figure 2:**
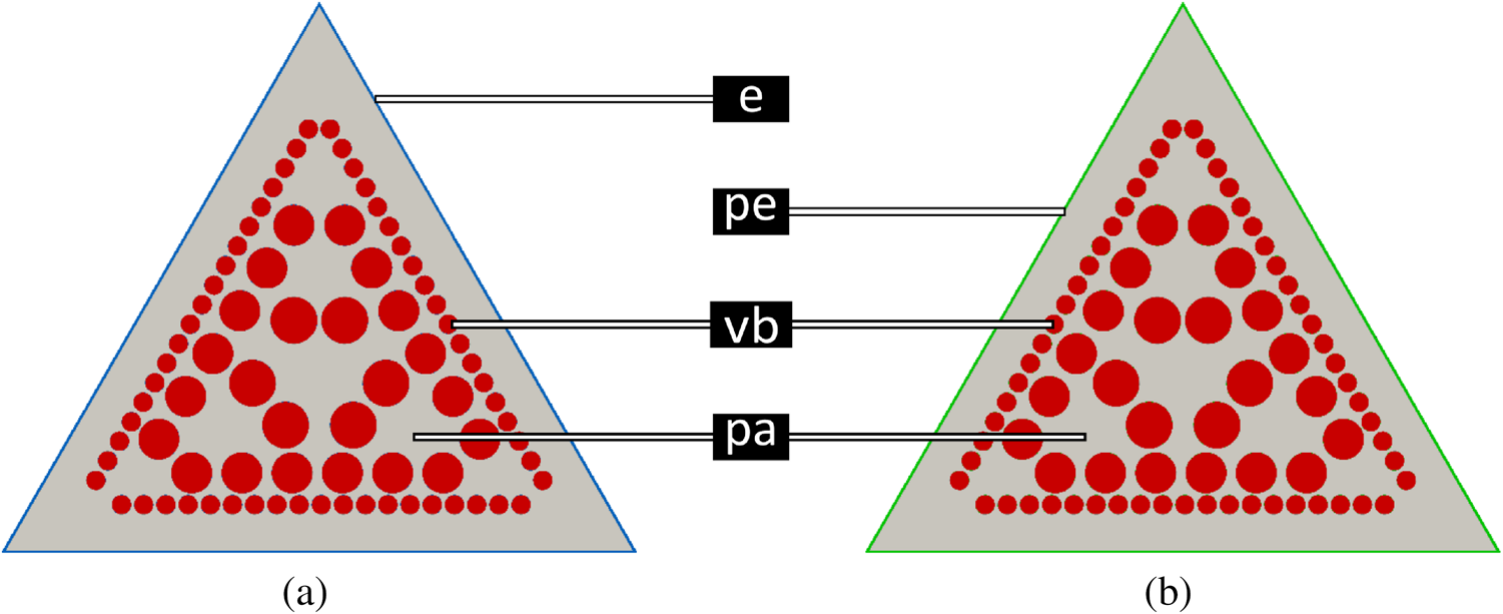
Initial configuration. Parenchyma and vascular bundles surrounded by (**a**) an epidermis ($E_{\text {norm}}=0.00111$) and (**b**) a peridermal covering tissue ($E_{\text {norm}}=0.01111$), respectively. For a non-lignified epidermis, both the torsional and flexural rigidity are comparatively low. If a corky periderm is formed, the flexural rigidity is increased by $\approx 14 \%$ and so is the torsional rigidity increased by $\approx 46\%$. This leads to a decrease in the twist-to-bend ratio by $\approx 24\%$. Abbreviations and colour code: e: epidermis (blue), pa: parenchyma (grey), pe: periderm (green), vb: vascular bundles (red).

During their ontogeny, plants react to increasing bending forces triggered by continuous growth of stem length and the formation of top loads such as flowers, seeds and fruits. An increase of their resistance against bending forces can be achieved by an increase of their flexural rigidity along the principal axes, i.e. increasing $D_{\text {min}}$. For a better understanding of this effect, we introduce a simplified model that is related to *C. pendula* and that measures flexural and torsional rigidity. As a first approach, we describe the tissue arrangement in the cross-section of *C. pendula* in terms of a distribution of circles (vascular bundles) in an equilateral triangular reference domain $\Omega$ additionally filled with parenchyma and surrounded by an epidermis (see Fig. 2a). The contribution to the cross-sectional area of the vascular bundles ($\approx 29\%$) and the parenchyma ($\approx 52 \%$) and the thickness of the epidermis is selected to match the distribution of mass in the total cross-sectional area of *C. pendula*.

For the computation of the rigidities, we normalise the elastic moduli ($E_{\text {est}}$) of all contained materials with respect to the elastic modulus of the sclerenchyma. This means that we set $E_{\text {norm}}=1$ for the sclerenchyma and obtain the normalised elastic moduli of the other materials by scaling accordingly (see Table 1). Furthermore, we consider the ratio between normalised elastic moduli of the stiffest and the most elastic material contained in the cross-section. As torsional and flexural rigidity are almost exclusively determined by these two materials, they are referred to as the mechanically decisive materials. Accordingly, the ratio between mechanically decisive materials is denoted by $\mu$ in the following.

We assume a constant Poisson’s ratio $\nu$ for the materials involved and compute the normalised torsional modulus $G_{\text {norm}}$ as

$$\begin{aligned} G_{\text {norm}}=\frac{E_{\text {norm}}}{2(1+\nu )} \end{aligned}$$

for a given elastic modulus $E_{\text {norm}}$. The assumption of a constant Poisson’s ratio is reasonable as the value range $\nu \in [0.2,0.5]$ is typical for many plant axes^28^ and, thus, a change in $\nu$ among the materials is negligible for our model. In the following, we set $\nu =0.35$ for *C. pendula*.

### Reinforcement without fibres: from epidermis to periderm

In this section, we compare the effect of a single-layered epidermis and a multi-layered corky periderm on the twist-to-bend ratio of the triangular cross-section (see Fig. 2). In this configuration and with respect to the normalised elastic modulus of the epidermis ($E_{\text {norm}}=0.00111$), the torsional rigidity ($D_{z}\approx 0.00013$) and the flexural rigidity ($D_{\text {min}} \approx 0.00118$) are comparatively low and so is the twist-to-bend ratio ($\frac{D_{\text {min}}}{D_{z}}\approx 9.37$) (see Fig. 2a). The resistance against bending forces and, hence, the flexural rigidity increases by the formation of a ring of corky periderm ($E_{\text {norm}}= 0.01111$). This formation of a closed ring of strengthening tissue, however, simultaneously increases the flexural rigidity ($D_{\text {min}}\approx 0.00134$) and the torsional rigidity ($D_{z}\approx 0.00019$) impeding the plant’s property of being able to twist easily and, moreover, decreasing its twist-to-bend ratio to ($\frac{D_{\text {min}}}{D_{z}}\approx 7.05$).

### Reinforcement by sclerenchyma fibre strands

**Figure 3:**
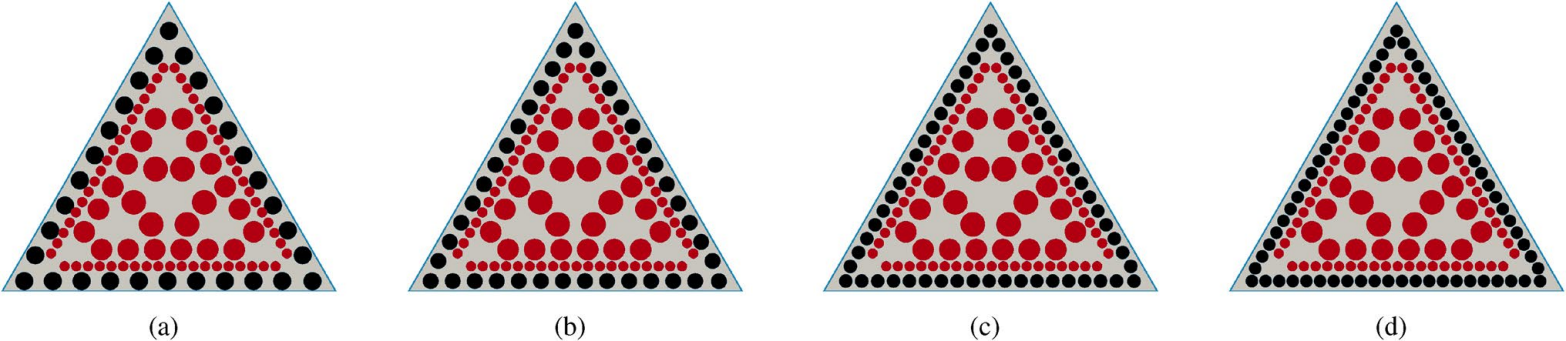
Reinforcement by fibre strands. From (**a**) to (**d**), the number of reinforcing (sclerenchyma) fibre strands increases whereas their total cross-sectional area remains constant. If the number is too high, single fibre strands become connected (**d**), thereby changing the torsional rigidity drastically and, thus, the procedure is stopped before this occurs. Colour code: epidermis (non-lignified): blue, parenchyma: grey, sclerenchyma fibres: black, vascular bundles: red.

In addition to the above-mentioned reinforcement of the epidermal tissue, fibre reinforcement is extremely common in plant axes. The cross-section of *C. pendula* shows individual sclerenchyma strands in the periphery directly under the epidermis (Fig. 1). Therefore, we additionally incorporated sclerenchyma fibre strands ($E_{\text {norm}}=1$) into the parenchyma ($E_{\text {norm}}=0.00044$) of the cross-section from Fig. 2a. We have developed a model to describe the effect of fibre reinforcement. The mechanically decisive materials for this experiment are parenchyma and sclerenchyma. The ratio $\mu$ between their elastic moduli is given by $\mu =0.00044$ . We fix the proportion of the sclerenchyma ($\approx 17\%$; see Table 1) in the total cross-sectional area. Further, we consider a distribution of fibre bundles (sclerenchyma) around the inner boundary $\partial \Omega$ of $\Omega$ with circular cross-sections centred at a fixed distance to $\partial \Omega$. Starting with 6 fibre strands for each side of the triangle we refine the distribution, such that a higher number of fibre strands is used in each step of the refinement, see Fig. 3. The proportion of the fibre bundles in the total cross-sectional area is fixed during the whole process. The arrangement and the total cross-sectional area of the fibre strands are now determined by the structure of the *Carex* ground tissue, see Fig. 1, and, hence, the number of fibre bundles is the only free variable in this model. The procedure is stopped before single fibre strands become connected, as such a closed ring of sclerenchyma would immediately (markedly) increase the torsional rigidity and thus decrease the twist-to-bend ratio.

For simplicity, circular cross-sectional geometries of the fibre bundles are taken, as circular cross-sections exhibit high torsional rigidity and, therefore, any other cross-sectional geometries of the fibre strands is likely to amplify the effect of decreasing torsional rigidity while increasing the number of fibre bundles.

**Figure 4:**
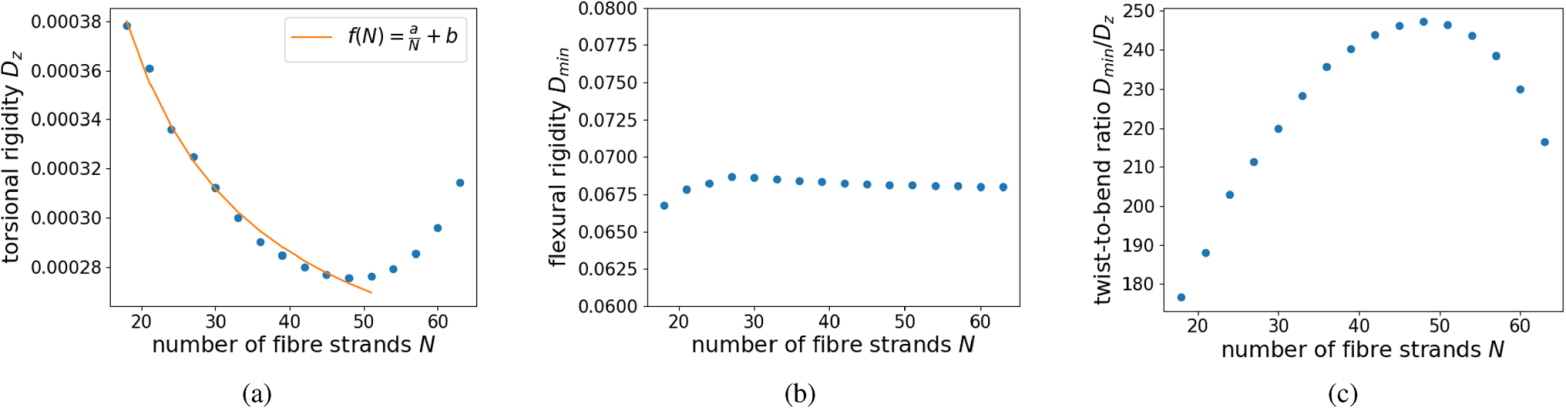
Reinforcement by sclerenchyma fibre strands. Trend of (**a**) torsional rigidity, (**b**) flexural rigidity and (**c**) twist-to-bend ratio. The ratio $\mu$ between the elastic moduli of the parenchyma and sclerenchyma is $\mu =0.00044$. Sclerenchyma and parenchyma are the mechanically decisive materials for this experiment. By increasing the number of fibre strands used for reinforcement, torsional rigidity first decreases linearly, see (**a**), whereas the flexural rigidity remains nearly constant during the procedure, see (**b**). The twist-to-bend ratio first increases nearly linearly reaching a maximum for $N =49$. For more than 49 fibre bundles, the distance of single fibre bundles is too small and the torsional rigidity is caused to increase again, whereas the twist-to-bend ratio is reduced by this effect, see (**a**) and (**c**). The numerically computed values of the torsional rigidity are further interpolated using a first order polynomial regression (orange line). For up to 51 fibre strands, the torsional rigidity behaves as $f(N)= \frac{a}{N}+C$, see (**a**), where $C=2.1\times 10^{-4}$ and $a=0.0031$. The R-squared value for the polynomial regression is 0.99.

The numerical experiments show that, by increasing the number of fibre strands, the torsional rigidity $D_{z}$ initially decreases when the flexural rigidity remains nearly bounded and, thus, the twist-to-bend ratio increases (see Fig. 4). A regression analysis for torsional rigidity up to 51 fibre strands shows that $D_{z}$ is given as a function of the number of fibre bundles *N*, with

$$\begin{aligned} D_{z}(N)= \frac{a}{N}+C \end{aligned}$$

for constants $a,C \in {\mathbb {R}}$. Therefore, increasing the number of fibre bundles initially decreases the torsional rigidity scaling as $N^{-1}$ in dependence on the number of fibre bundles with asymptote *C*. This decline in the torsional rigidity is driven by a decreasing amplitude of Prandtl’s stress function around fibre strands, see Fig. 6c and d. Because of the boundedness of the flexural rigidity, the twist-to-bend ratio increases showing a similar asymptotic behaviour and scaling as *N* in dependence on the number of fibre strands.

However, when the distance between neighbouring fibre strands becomes very small, even when the fibre strands are not yet connected, see (a) in Fig. 6, the ring-like structure of the fibre arrangement causes the torsional rigidity to increase again leading to a decrease in the twist-to-bend ratio. This occurs because the gradient of $\phi$ in the space between the fibre strands increases as the distance between fibre strands decreases, similar to the behaviour of a Neumann sieve^29^. Thus, the value of $\phi$ in the inner part of the cross-section is raised, see (e) and (f) in Fig. 6, resulting in an increase of the torsional rigidity when the number of fibres increases beyond 49 (see Fig. 4a) even if no closed ring is formed yet.

Since this increase is integrated over a large domain (the inner part of the triangle) the effect becomes more dominant for smaller distances of neighbouring fibre strands and therefore leads to an increase in the torsional rigidity, see Fig 4a. Thus in the setting described above, torsional rigidity and the twist-to-bend ratio reach an optimum for 49 fibre strands, see Fig. 4a,c. This characterises the number of fibre bundles *N* as a design variable in an optimisation problem in order to maximise the twist-to-bend ratio and simultaneously minimise the torsional rigidity.

### Reinforcement by collenchyma fibre strands

Following the previous section, we can now reasonably investigate the dependency of the optimal number of fibre strands needed to increase the twist-to-bend ratio on the ratio $\mu$ between the elastic moduli of the mechanically decisive materials involved. To do so, we replace the sclerenchyma fibres ($E_{\text {norm}}=1$) shown in Fig. 3 by collenchyma fibres ($E_{\text {norm}}=0.05555$), see Table 2. Since now parenchyma ($E_{\text {norm}}=0.00044$) and collenchyma are the decisive materials, this leads to a ratio $\mu \approx 0.008$. We can again carry out the experiment shown in Fig. 3 but with collenchyma fibres instead of sclerenchyma fibres and with the normalised elastic modulus $E_{\text {norm}}=0.05555$ for the collenchyma fibres. The elastic moduli of the other materials involved (epidermis, parenchyma, vascular bundles) remain the same.

**Figure 5:**
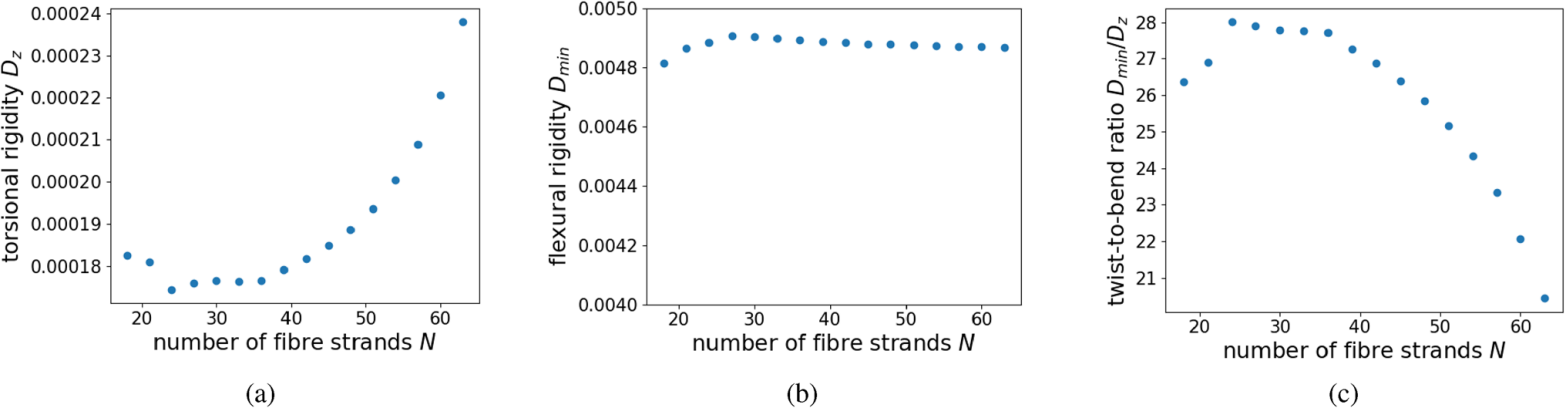
Reinforcement by hypothetically occurring collenchyma fibre strands. Trend of (**a**) torsional rigidity, (**b**) flexural rigidity and (**c**) twist-to-bend ratio. The ratio $\mu$ between the elastic moduli of the parenchyma and collenchyma is $\mu =0.008$. The collenchyma and parenchyma are the mechanically decisive materials. By increasing the number of fibre strands used for reinforcement, the torsional rigidity first decreases nearly linearly, see (**a**), whereas the flexural rigidity remains nearly constant during the procedure, see (**b**). The twist-to-bend ratio first increases nearly linearly reaching a maximum for $N=24$. For more than 24 strands, the torsional rigidity is caused to increase again, whereas the twist-to-bend ratio is reduced by this effect, see (**a**,**c**).

Again, we find that up to a certain number of collenchyma strands, the torsional rigidity decreases linearly, whereas the flexural rigidity remains nearly constant, see Fig. 5a,b. However, now that the ratio $\mu$ is lower, the number of fibre strands that is optimal in order to decrease torsional rigidity and to increase the twist-to-bend ratio, is reduced noticeably to 24 and,–after a slight drop–the twist-to-bend ratio remains nearly constant up to 36. For more than 24 collenchyma strands, we can see the same effect as above, namely the torsional rigidity increases again and, hence, the twist-to-bend ratio decreases, see Fig. 5a,c.

This experiment, in which the sclerenchyma fibres are replaced by collenchyma fibres, illustrates the effect of the ratio $\mu$ between the elastic moduli of the mechanically decisive materials on the twist-to-bend ratio and on the optimal number of fibre strands included in fibre reinforcement. As a preview to the discussion, we can conjecture that the ratio $\mu$ plays a role in the sense that, for the higher ratio $\mu$, the optimal number of fibre bundles in order to increase the twist-to-bend ratio is lower and vice versa. When collenchyma fibres are incorporated instead of sclerenchyma fibres, $D_{\text {min}}$ decreases by a factor of approximately 13.5 resulting in a markedly reduced resistance against bending forces.

**Table 3:** Summary of the simulation results.

decisive materials	$\mu$	flexural rigidity	torsional rigidity	twist-to-bend ratio
Parenchyma/epidermis	0.4	reference value	reference value	reference value
Parenchyma/periderm	0.04	increase $\approx 14 \%$	increase $\approx 46 \%$	decrease $\approx 24 \%$
Parenchyma/sclerenchyma*	0.00044	increase $\approx 5600 \%$	increase $\approx 112 \%$	increase $\approx 2536 \%$
Parenchyma/collenchyma**	0.008	increase $\approx 315 \%$	increase $\approx 34 \%$	increase $\approx 198 \%$

The mechanical effect of various changes of the structural reinforcement is given as a percentage increase or decrease with reference to the initial configuration (= reference value). Figure 2a shows the initial configuration in terms of a triangular cross-section comprising of parenchyma with embedded vascular bundles surrounded by an epidermis. $\mu$ is the ratio of estimated elastic moduli (*E*_est_) of the decisive materials (see Table 2). The percentage values inherent to the fibre reinforcements are computed with respect to the optimal number of fibre strands. namely, *49 sclerenchyma strands and **24 collenchyma strands (see Figs. 4, 5).

**Figure 6:**
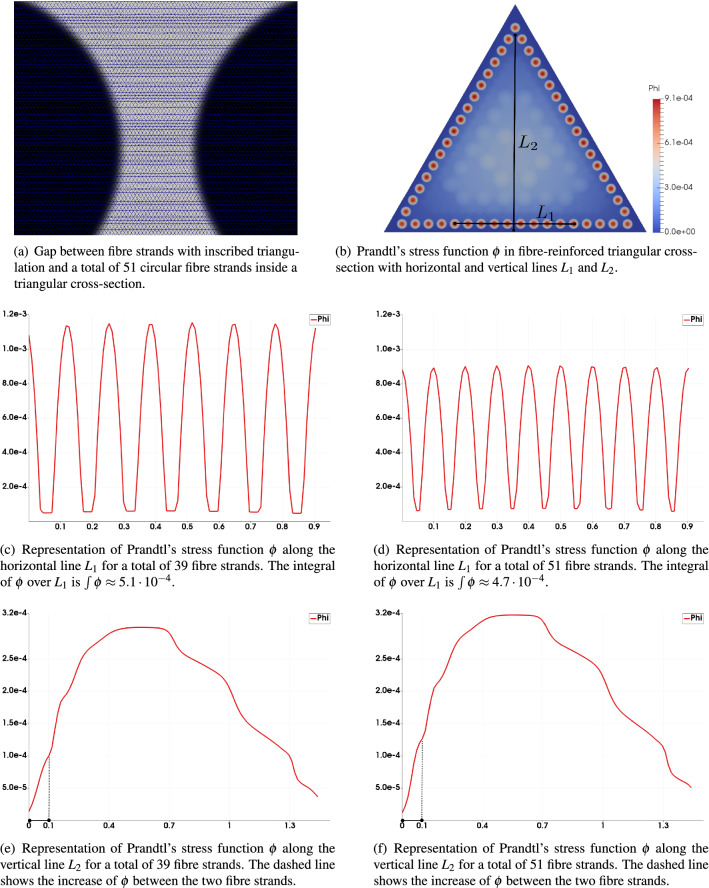
Local representation of Prandtl’s stress function. For different numbers of fibre strands, the stress function $\phi$ is plotted along horizontal and vertical lines, $L_{1}$ and $L_{2}$, in (**b**) and thereby two opposing effects are illustrated. By increasing the number of fibre strands but by fixing their total area, $\phi$ decreases within the number of fibre strands, see (**c**) and (**d**), but increases in the inner part of the cross-section, see (**e**) and (**f**). This because, as the distance between the fibre strands becomes smaller, the gradient of $\phi$ between two fibre strands is increased and, therefore, the value of $\phi$ in the inner part of the cross-section is raised. This effect becomes dominant from a certain number of fibre strands and, thus, increases the torsional rigidity. Further, the impact of these effects on the torsional rigidity depends on the ratio $\mu$ between the elastic moduli of the two mechanically decisive materials. For high ratios $\mu$, the impact of the effect in (**e**) and (**f**) is more pronounced, whereas for low ratios, the impact of the effect in (**c**) and (**d**) is more intense. The fineness of the triangulation is sufficiently high, so that even small gaps between fibre strands can be resolved, see (**a**).

## Discussion

When embryophytic plants colonised the land and lost the buoyancy of surrounding water around 470 million year ago during the mid-Ordovician, they faced entirely different mechanical constraints. These concerned, in particular, their anchorage (root system) and the mechanical loads on their upright aerial parts (stems with branches and leaves). Stems, roots and leaves had evolved as early as the mid Devonian (ca. 390 million years BP) and the first forests of tall trees existed by the late Devonian (ca. 370 million years BP)^30,31^. Since then, evolutionary processes have shaped stem and root form and internal structure enabling plants to cope with the various mechanical loads that act upon them in their diverse environments. A plethora of evolutionary adaptations can be observed in aerial stems, especially with regard to bending and torsional loading, which represent the predominant load cases in plants with self-supporting upright stems. In addition to experimental analyses, theoretical considerations including analytical and numerical simulations can help to decipher the complex interplay between form, structure and mechanical properties of the involved plant tissues^24,32–35^.

In this article, we present our finite element analysis involving a triangular cross-section consisting of parenchyma with scattered embedded vascular bundles surrounded by an epidermis (see Fig. 2a). In contrast to real experiments, computer experiments allow one particular variable to be altered and the resulting effect to be quantified precisely. By replacing the epidermis with a periderm or by including additional fibre strands with variable numbers and/or mechanical quality, we can quantify the mechanical effect of structural reinforcement on the flexural and torsional rigidity and thus the twist-to-bend ratio of plant axes. The biological model that we have used for our simulation is the triangular cross-section of the flower stalk of *C. pendula*, which shows the so far highest twist-to-bend ratio measured in plant stems. The long, drooping stem attains lengths of up to 2.5 m and bears apically pendulous inflorescences that develop into a relatively heavy multiple fruit. The presented approach gives us the possibility to give answers to our scientific question “To what extent do individual tissues such as fibres, vascular bundles, epidermis and parenchyma contribute to the flexural rigidity, the torsional rigidity and thus to the twist-to-bend ratio of a triangular plant axis?” Table 3 gives an overview on the results, which will be discussed in detail in the following.

### From epidermis to periderm

The outermost single cell layer of plants is called the epidermis, the main function of which is to protect the plant. This primary covering with an elastic modulus of $E \approx 50$ MPa can support tensile loads. Woody stems can also produce a secondary protective covering, called the periderm, which replaces the epidermis locally or globally. The periderm has an elastic modulus of $E \approx 500$ MPa (unpublished data). A triangular cross-section comprising of parenchyma and vascular bundles surrounded by an epidermis (see Fig. 2a) shows relatively low values of the flexural and torsional rigidity. If the epidermis is replaced by a periderm, which forms a closed ring (see Fig. 2b), the flexural rigidity increases by $\approx 14 \%$ and the torsional rigidity increases by $\approx 46\%$. This results in a decrease of the twist-to-bend ratio of $\approx 24\%$ in cases with the formation of periderm (see Table 3).

The cactus *O. ficus-indica* shows pronounced formation of periderm at its stem base at which the greatest bending moments occur, because of the long lever arm, and high torsional forces can appear because of the firm clamping by rooting in the soil. In addition, increased periderm formation can be found at the narrowed cross-sections of the transitions between its branches, at which point an increased risk of breakage through bending or torsion forces by wind loads and passing animals exists^7^. If exposed to moderate wind, tall and shrubby cacti do not become streamlined, but start to oscillate. Under these conditions, high torsional rigidity together with high bending rigidity is advantageous, because it prevents damage and breakage. We therefore hypothesise that periderm formation takes place at mechanically highly stressed or damage-prone areas (e.g. at the basis of the entire plant, the transition between the branches and the main stem and the branches). Periderm formation might be triggered by tiny injuries arising from overcritical local mechanical stress. If the cacti are exposed to heavy wind, single branches are torn off or the whole plant is uprooted.

The formation of an outer secondary protection tissues (secondary cortex = periderm) covering the epidermis in older ontogenetic stages was probably an evolutionary important step allowing an increase in stem girth because of the production of secondary xylem and phloem by secondary vascular growth. Some of the first fossil records of secondary cortex originate from the late Devonian/early Carboniferous members of the progymnosperms, lycopsids and pteridosperms^31,34–36^. Periderm formation not only allows secondary stem growth, which increases girth, through the simultaneous growth of an outer secondary protection layer, but also may take over mechanical functions^36–38^ and ensure repair after injuries^7^.

Whereas, in extant perennial arborescent growth forms, the mechanical properties and stability are governed by secondary wood (= secondary xylem), this was not the case in a group of important forest-forming plants during the late Devonian and Carboniferous: the arborescent lycopsids. In this group with scale trees (genus: *Lepidodendron*) and seal trees (genus: *Sigillaria*), the periderm, which contributed over $90\%$ of the stem volume, was by far the mechanically dominant tissue^31,32,36,39^. Peripheral stiffening structures were the first specialised stabilising tissues evolved in early land plants. After the colonisation of the land by early plant genera with turgor-stabilised stems, specialised collenchymatous or sclerenchymatous stiffening tissues, the so-called hypodermal steromes lying directly underneath the epidermis, evolved as early as the lower Devonian. These hypodermal steromes allowed not only the colonisation of dryer habitats, but also a marked increase in plant height^30–33,36^.

### Reinforcement by sclerenchyma fibre strands

As documented in Table 2, sclerenchyma fibres are the stiffest material in our simulation with an elastic modulus of $E \approx 45$ GPa. Thus, we selected it as our reference material ($E_{\text {norm}}=1$). We added fibre strands to the initial configuration, with the total area of the strands remaining constant. In this simulation, we investigated the effect of an increasing number of individual peripheral sclerenchyma strands in a parenchymatous matrix with vascular bundles being surrounded by an epidermis (see Fig. 3). The flexural rigidity remained almost constant independent of the number of sclerenchyma strands, namely between 18 and 63 strands (see Fig. 4b). In contrast, the torsional rigidity decreased nearly linearly from 18 to 49 strands. From 50 to 63 strands, it increased again to $\approx 140\%$ of the initial value (see Fig. 4a). Since the flexural rigidity remained almost constant, the twist-to-bend ratio initially increased almost linearly up to an optimum at 49 strands and then decreased to $\approx 2200\%$ of the initial value (see Fig. 4c). Compared with the initial configuration, the addition of 49 sclerenchyma strands led to enormous increases in the flexural and torsional rigidity and the twist-to-bend ratio (see Table 3).

This optimum of $N = 49$ strands found in the simulation corresponds well with the average value of $49.40 \pm 7.83$, which is calculated from the numbers of strands of the apical internode ($42.80 \pm 4.31$) and the more basal internode ($56.00 \pm 3.87$, see Table 1) of *C. pendula*^2^. The clear optimum of the U-shaped curve (see Fig. 4a) is of interest in so far that sclerenchyma fibres are energetically highly costly for plants because of their extremely thick secondary cell walls that are impregnated with the macromolecule lignin. For annual plants, in particular, this is an expensive investment in a strengthening tissue that is, however, highly rigid. Since sclerenchyma fibres are dead cells, no further investment is required once they have been formed. This is especially advantageous for perennial plants.

With twist-to-bend ratios of up to 403, the internodes of *C. pendula* show the highest values ever measured in plant axes^2^. The high flexural rigidity and low torsional rigidity of the flower stalk are particularly advantageous under dynamic wind loads. The high torsional flexibility allows the streamlining of the stalks together with the apical pendulous inflorescences or multiple fruits. In summer, the flower stalks have to bear these additional heavy fruits without bending down to the ground. Our simulations show that flexural rigidity cannot be achieved by the formation of additional sclerenchyma strands, as the number is already in the range of the optimum. However, a further increase in flexural rigidity is ensured by the rigid leaf sheaths that enclose large parts of the flower stalk. Since the leaf sheaths are not firmly attached to the stem, they are unlikely to have a strong negative effect on torsional flexibility.

### Reinforcement by collenchyma fibre strands

Collenchyma fibres are living cells that possess a vacuole and a thick non-lignified primary cell wall. Therefore, their elastic modulus is turgor-dependent. With an elastic modulus of $E \approx 2.5$ GPa, collenchyma fibres belong to the strengthening tissues ($E_{\text {norm}}=0.05555$) and are often found in still-growing shoots and leaves. From an energy point of view, collenchyma fibres are much cheaper to build than sclerenchyma fibres, because neither thick secondary cell walls nor the macromolecule lignin need to be formed in the former. Furthermore, collenchyma fibres have the advantage that they are still able to grow or to be stretched. On the other hand, as living cells, they always consume physiological energy and hence, are predominantly found in young growing tissues and in annual plants or plant organs.

The square flower stalks of the perennial *L. cardiaca* show pronounced strands of collenchyma fibres at the four corners and small strands in the middle of the four sides. Dependent on the height above ground and increasing age, the cross-sectional percentage area of collenchyma decreases from $8.61\%$ to $6.18\%$. With increasing age and mass attributable to the formation of flowers and heavy fruits, the peripheral lignified vascular tissues with a percentage cross-sectional area increasing from 15.55% to $21.00\%$ increasingly take over the mechanical support of the stem. Analyses have demonstrated that the area sum of the vascular tissues and parenchyma exhibit moderate positive allometric scaling, whereas the collenchyma shows clear negative scaling^8^.

In the circular leaf stalks of *C. bicolor* ‘Candyland’, the cross-sectional percentage area of the $\approx 40$ peripheral collenchyma strands has a value of $3.9 \pm 0.7\%$. The cross-sectional area of the parenchyma and the lignified elements are $89.0\pm 3.3\%$ and $1.2 \pm 0.4\%$, respectively. Parenchyma and collenchyma are turgor-dependent. Together, they form a doubly secured mechanical system that is sensitive to drought stress. The decrease of flexural rigidity and, thus, the wilting of the leaf stalk are the result of a turgor-loss-induced decrease of the elastic moduli of both the collenchyma fibres and the parenchyma cells^5^. As a withered leaf stalk cannot be restored to its healthy positioning, even with sufficient water support, the evolution of a redundant mechanical system to maintain the flexural rigidity of the plant, in particular, is of great advantage for selection.

In the case of *C. pendula*, the replacement of sclerenchyma fibres by collenchyma fibres showed an increase of flexural rigidity by only $\approx 315 \%$ instead of $\approx 5600 \%$ (see Table 3). Reinforcement by collenchyma fibre strands would be much too weak mechanically to support the stalk. Moreover, even the addition of an optimal number of sclerenchyma strands is probably insufficient. During autumn, in particular, when the flower stalk has a heavy top load of fruits, additional leaf sheaths increase the flexural rigidity of the overall system.

## Conclusion

We have shown here that the ratio of the elastic moduli of the materials, which are decisive for the mechanical performance of the entire plant axis ($\mu$), plays a crucial role in plant stems. Reinforcements generally increase the flexural and torsional rigidities in a triangular cross-section composed of a parenchymatous matrix with embedded scattered vascular bundles surrounded by an epidermis. Closed ring-shaped reinforcement of the epidermal tissue (e.g. a periderm) leads to a considerable increase in torsional rigidity and a moderate increase in flexural rigidity in areas of the plants that are mechanically heavily loaded or at risk of damage. Therefore, epidermal reinforcements decrease the twist-to-bend-ratio. Fibre reinforcement noticeably increases the flexural rigidity and moderately increases the torsional rigidity of the entire plant axis. The flexural rigidity is almost independent of the number of fibre strands, whereas the torsional rigidity and thus the twist-to-bend ratio is a function of strand numbers. Obviously, torsional rigidity is the key factor for changing the twist-to-bend ratio through structural reinforcements. The evolution of structural reinforcements including cells with walls strengthened by lignin was a prerequisite for land plants to be able to colonise terrestrial habitats and successfully enabled them to face notably different mechanical constraints compared with those experienced in an aquatic environment. As outlined above, the primary (e.g. hypodermal sterome) and secondary (e.g. wood, cortex, periderm) strengthening tissues allowed plants not only to increase markedly in height, but also to colonise more and more hostile (dryer) habitats leading to the plethora of plant life forms that we know from extant and fossil flora^30–32,36^.

### Research involving plants

The Botanic Garden of the University of Freiburg acts in accordance with the CBD, the Nagoya Protocol and the Convention on the International Trade in Endangered Species (CITES) and is member of IPEN (International Plant Exchange Network). The IPEN-Number of the experimental *Carex pendula* plants is XX-0-FB-1420.

## Supplementary Information

Supplementary Information.

## Data Availability

This work includes no experimental data. A C++-implementation including the computation of rigidities is available in the supplementary material.
